# Transcriptome Profile Analysis of Breast Muscle Tissues from High or Low Levels of Atmospheric Ammonia Exposed Broilers (*Gallus gallus*)

**DOI:** 10.1371/journal.pone.0162631

**Published:** 2016-09-09

**Authors:** Bao Yi, Liang Chen, Renna Sa, Ruqing Zhong, Huan Xing, Hongfu Zhang

**Affiliations:** State Key Laboratory of Animal Nutrition, Institute of Animal Sciences, Chinese Academy of Agricultural Sciences, Beijing, 100193, China; Kunming University of Science and Technology, CHINA

## Abstract

Atmospheric ammonia is a common problem in poultry industry. High concentrations of aerial ammonia cause great harm to broilers' health and production. For the consideration of human health, the limit exposure concentration of ammonia in houses is set at 25 ppm. Previous reports have shown that 25 ppm is still detrimental to livestock, especially the gastrointestinal tract and respiratory tract, but the negative relationship between ammonia exposure and the tissue of breast muscle of broilers is still unknown. In the present study, 25 ppm ammonia in poultry houses was found to lower slaughter performance and breast yield. Then, high-throughput RNA sequencing was utilized to identify differentially expressed genes in breast muscle of broiler chickens exposed to high (25 ppm) or low (3 ppm) levels of atmospheric ammonia. The transcriptome analysis showed that 163 genes (fold change ≥ 2 or ≤ 0.5; *P*-value < 0.05) were differentially expressed between Ammonia25 (treatment group) and Ammonia3 (control group), including 96 down-regulated and 67 up-regulated genes. qRT-PCR analysis validated the transcriptomic results of RNA sequencing. Gene Ontology (GO) functional annotation analysis revealed potential genes, processes and pathways with putative involvement in growth and development inhibition of breast muscle in broilers caused by aerial ammonia exposure. This study facilitates understanding of the genetic architecture of the chicken breast muscle transcriptome, and has identified candidate genes for breast muscle response to atmospheric ammonia exposure.

## Introduction

Ammonia (NH_3_) as a toxic gas is recognized as one of the most prominent contaminants in buildings housing broiler chickens for many years [1]. Ammonia mainly originates from the decomposition of the nitrogen-containing excretion from the kidneys and the gut of birds [2,3]. Atmospheric ammonia is a common problem in poultry houses and longtime exposure to ammonia can adversely affect bird performance and profit [4–6].

Abundant reviews and research articles have shown that high concentrations of atmospheric ammonia (higher than 25 ppm) can be detrimental to bird health and performance [7–11]. In these experiments, poultry are often exposed to levels of over 50 ppm ammonia or more, sometimes even excessively high up to 100~200 ppm [4,10,12–14]. This sort of high levels of ammonia is in fact unrealistic in modern commercial broiler industry. In production practice, throughout the whole production period, the typical range of mean aerial ammonia concentrations in poultry houses were between 5 and 30 ppm [15,16]. It has been suggested that ammonia should not exceed 25 ppm in poultry houses [1]. Furthermore, the recommended exposure limit for ammonia of 25 ppm is based on human safety rather than animals' consideration. However, until now, plenty of research have demonstrated that the levels of 25 ppm ammonia have adverse effects on the production and health of poultry. Caveny et al. found that birds exposed to 25 ppm of ammonia for 42 days resulted in decreased feed efficiency [17]. Anderson et al. provided the evidence that ammonia at concentrations of 25 ppm could impair the mucous flow and ciliary action of respiratory tract in broilers [18]. Different research team have shown that body weights of broilers were reduced varying degrees at 25 ppm ammonia treatment [10,14,19,20].

To date, numerous studies mainly focus on the impact of gaseous ammonia on respiratory system and performance in broilers [21,22]. Research on the impact of ammonia on breast muscle tissue of broiler chickens are scarce. Breast meat production and yield is essential important for consumers worldwide. The profitability of broiler industry is largely determined by the increasing proportion of prime parts in the carcass, mainly breast meat [23,24]. Charles and Payne revealed that ammonia exposure bring about a significant increase in breast blisters on broilers [25]. It has been shown that broilers exposed to atmospheric ammonia result to a reduction in body weight and meat yield of broilers, but the possible mechanisms not to be involved [10].

Based on previous research, the present study investigated the effect of ammonia at levels of 25 ppm on breast muscles, and utilized high throughput RNA sequencing technology to deeply explore the molecular mechanisms and candidate genes related to ammonia exposure. Therefore, the objectives of the work described in this paper were to determine the effect of 25 ppm atmospheric ammonia exposure on broiler performance, gene expression changes and molecular mechanisms associated with breast muscle development underlying aerial ammonia exposure. This study provides a theoretical basis for reducing ammonia concentrations, improving air quality and animal welfare in broiler industry.

## Materials and Methods

### Ethics Statement

All procedures were authorized by the Institutional Animal Care and Use Ethics Committee of Chinese Academy of Agricultural Sciences and all broiler treatments were carried out in accordance with the Regulations for the Administration of Affairs Concerning Experimental Animals of the State Council of the People's Republic of China. The minimum number of broilers was used and every effort was made to reduce their discomfort and stress.

### Experimental Animals and Ammonia Exposure Treatments

A total of ninety-six 1-day-old male Arbor Acres (AA) broiler chicks were purchased from a commercial hatchery in Beijing (Beijing Arbor Acers Broiler Co., Beijing, China). All birds were housed in individual wire-bottom cages in an environmentally controlled room under standard brooding practices. Then, 21-day-old AA broilers with close body weights were divided into two groups for Ammonia3 (control group) and Ammonia25 (treatment group), so that the birds with insignificant mean weight for each group were exposed to different levels of aerial ammonia. Each group had 8 replicates, 6 birds per replicate. Ammonia exposure chambers were two separate 4500 × 3000 × 2500 mm (length × width × height) sealed units. Broilers in the control group were kept at 3 ± 3 ppm ammonia during the experimental period. Broilers in the treatment group were raised in a separate chamber for the same period, and the concentration of ambient ammonia was kept at 25 ± 3 ppm. During the entire experiment, the concentration of ammonia in both chambers was monitored using the instrument LumaSense Photoacoustic Field Gas-Monitor Innova-1412 (Santa Clara, CA, USA) [21]. Birds were reared in two programmed artificial climate chambers and fed a corn-soybean basal diet. The diet during the experiment was formulated to achieve the National Research Council (NRC, 1994) recommended requirements for all nutrients (S1 Table). Body weight and feed consumption were recorded weekly for feed conversion ratio evaluation. This study was conducted in the State Key Laboratory of Animal Nutrition, Changping District, Beijing, China.

### Sample Collection

At day 42, all birds were weighed after a 12 h-fasting (12 h food withdrawal) period. Production parameters (n = 48) including average daily feed intake, body weight gain and feed-conversion ratio were determined. To examine the slaughter performance, the chickens were euthanized by manual cervical dislocation and then exsanguinated for tissue sampling. Slaughter rate, half net carcass rate and whole net carcass rate were determined. Samples of breast muscles were quickly dissected and weighed, then rapidly frozen in liquid nitrogen, and stored at -80°C for further transcriptome (RNA sequencing) and qRT-PCR analyses.

### Total RNA Extraction and cDNA Library Construction

Breast muscles of the 4 randomly selected broilers (2 birds per group) were separately ground in frozen state in liquid nitrogen [26]. Total RNA of breast muscle tissue was isolated with TRIzol reagent (Invitrogen, USA) according to the manufacturer's instructions. The concentrations of RNA samples were measured using the NanoDrop 2000 (Nanodrop Technologies, Wilmington, DE). Agilent 2100 Bioanalyzer (Agilent Technologies, Santa Clara, CA) was utilized to assess the integrity of the total RNA (RIN number > 8.0). The total RNA with lowest quality was not used for further study.

Sequencing libraries were generated using the TruSeq RNA Sample Prep kit v2 (Illumina, San Diego, CA) following manufacturer's instructions. Briefly, mRNA was extracted from total RNA using oligo (dT) magnetic beads (Invitrogen, USA) and cleaved into short fragments of about 200 bases. These fragmented mRNAs were then used as templates for cDNA synthesis. The cDNAs were then PCR amplified to complete the library. After PCR enrichment, cDNA quantity and quality were assessed using a NanoDrop 2000 spectrophotometer (Nanodrop Technologies, Wilmington, DE) and Agilent 2100 Bioanalyzer (Agilent Technologies, Santa Clara, CA). After construction, the 4 cDNA libraries were normalized, as suggested by the manufacturer, to 10 nmol/μl using Tris buffer (10 mmol Tris-HCl, 0.1% Tween 20, pH 8.5).

### Transcriptome Sequencing and Bioinformatics Analysis

The paired-end high throughput transcriptome sequencing was performed on the Illumina HiSeq 2000 sequencing platform. The raw data generated from the Illumina Hiseq platform were filtered using a quality control analysis, clean reads were obtained by removing low quality reads (threshold quality, 20; threshold length, 50 bp) as well as reads containing adapter sequences, ploy-N and the sequencing primer from the raw data. At the same time, Q30, GC-content and sequence duplication level of the clean data were calculated. The clean reads were mapped to the *Gallus gallus* genome (ftp://ftp.ncbi.nlm.nih.gov/genomes/all/GCF_000002315.3_Gallus_gallus4.0/GCF_000002315.3_Gallus_gallus-4.0_genomic.gff.gz) in TopHat. Transcript abundance was normalized into FPKM (fragments per kilobase of exon model per million mapped reads). Differential expression of genes between groups was analyzed using the DEGseq R package. Genes with *P*-value < 0.05 and fold-change ≥ 2 or ≤ 0.5 were considered significant. The raw RNA sequencing data were deposited to the GEO database of NCBI (accession number: GSE85786).

### GO Annotation and KEGG Pathway Analysis of Differentially Expressed Genes

All of the differentially expressed genes that were significantly altered in breast muscle samples of ammonia-exposed broiler chickens were annotated using Gene Ontology (GO) annotation. After the GO annotation, the WEGO tool (http://wego.genomics.org.cn/cgi-bin/wego/index.pl) was employed to visualize the functional classification for the differentially expressed genes [27,28].

Database for Annotation, Visualization and Integrated Discovery (DAVID) (http://david.abcc.ncifcrf.gov/) and the Kyoto encyclopedia of genes and genomes (KEGG) database (http://www.genome.jp/kegg) [29,30] were used to classify differentially expressed genes in significantly overrepresented pathways and GO terms.

### Quantitative Real Time PCR (qRT-PCR)

To validate the RNA sequencing results, qRT-PCR was performed with the CFX96 Real-Time PCR Detection System (Bio-Rad, USA). Total RNA of breast muscle samples was isolated using Trizol Reagent (Invitrogen, USA) according to the manufacturer's instructions. RNA concentration was measured with a NanoDrop 2000 (Nanodrop Technologies, Wilmington, DE). cDNA used for qPCR was synthesized from the mRNA by reverse transcription reaction using the PrimerScrip^™^ RT reagent Kit (Takara, Japan). The primer sequences were synthesized in Sangon Biotech (Shanghai, China) based on the mRNA sequences obtained from the NCBI database. The 20 μl PCR reaction mixture contained 10 μl SYBR^®^ Fast qPCR Mix (2×) (Takara, Japan), 0.8 μl forward primer (10 μmol/L), 0.8 μl reverse primer (10 μmol/L), 2 μl cDNA template and 6.4 μl RNase-free H_2_O. The PCR reaction was set as follows: 95°C for 30 s; 40 cycles of 95°C for 5 s, 60°C for 10 s and melting curve analysis. The qPCR analysis was performed with three biological and technical replicates. The relative fold changes of gene expression were calculated according to the 2^−ΔΔCT^ method using *GAPDH* (glyceraldehyde-3-phosphate dehydrogenase) as an internal control. The primers used are listed in S2 Table.

### Statistical Analysis

In the statistical analysis, GraphPad Prism (v6.07) and JMP (version 10) programs were used. Significant differences were examined using one-way ANOVA and Student's *t*-test, and differences with *P* < 0.05 were considered statistically significant for all experiments.

## Results

### Effect of Atmospheric Ammonia on Performances of Broiler Chickens

In this study, during the entire experimental period (21 days), birds from Ammonia25 (treatment) group had 2.8% less slaughter rate (*P* < 0.05) and 31.8% less beast muscle ratio (*P* < 0.05) compared with chickens from Ammonia3 (control) group (Table 1). Weight gain and feed intake are key production parameters to assess animal growth performance. However, in this study, chickens from Ammonia3 and Ammonia25 groups were not significantly different (*P* > 0.05) in average daily gain (ADG), average daily feed intake (ADFI) and feed conversion ratio (FCR).

**Table 1:** The effects of atmospheric ammonia on growth performance and slaughter performance of 42-day AA broilers.

	3 ± 3 ppm	25 ± 3 ppm
Group	Ammonia3	Ammonia25
ADFI	158.42 ± 5.78	152.46 ± 7.69
ADG	99.41 ± 10.08	89.36 ± 12.32
FCR	1.64 ± 0.18	1.71 ± 0.23
Slaughter rate (%)	91.96 ± 0.35^a^	89.38 ± 1.73^b^
half net carcass rate (%)	85.74 ± 0.82	84.28 ± 1.46
Whole net carcass rate (%)	73.00 ± 0.86	72.34 ± 1.57
Beast muscle ratio (%)	23.61 ± 1.06^a^	16.11 ± 2.47^b^

ADFI, average daily feed intake; ADG, average daily gain; FCR, feed conversion ratio. Values ^a, b^ within a row indicate significant difference between groups at *P* < 0.05. Numbers are mean ± S.D.

### Transcriptome Profiles

**Fig 1:**
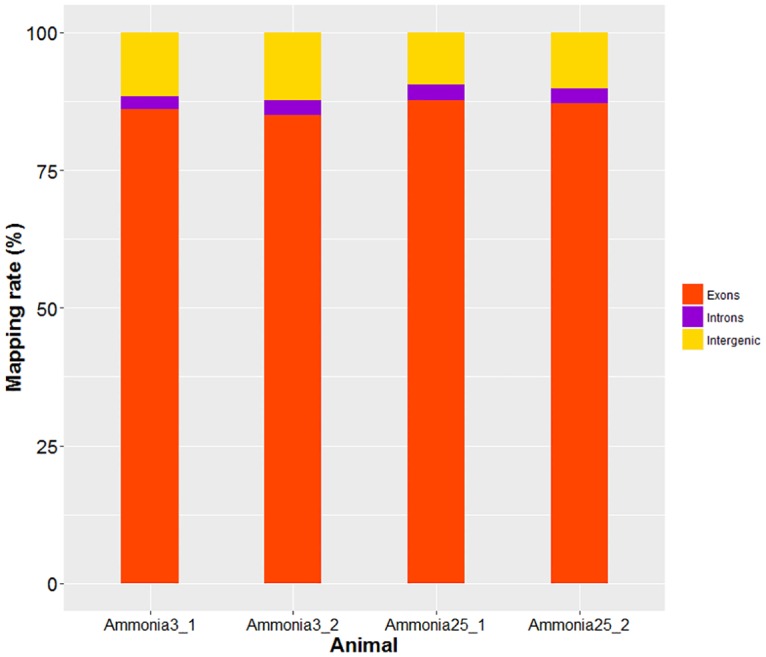
The percentage of reads mapped to exonic, intronic and intergenic regions.

High throughput RNA sequencing was used to systematic analyses of gene expressions of breast muscle tissue transcriptome with high or low levels of atmospheric ammonia exposure. The RNA sequencing of four breast muscle samples yielded around 319.3 million of raw paired-end reads. After quality control, high-quality reads of each sample ranging from 67 to 83 million, over 70% clean reads per sample were mapped to the reference genome and used for gene expression analysis (Table 2). Approximate 65% reads were aligned in a unique manner, and about 5% as multiple mapped manner. Among total mapped reads, the vast majority of which (85.05%~87.72%) fell into annotated exons, 9.45~12.24% was within the large intergenic territory, and only 2.33~2.83% was located in introns (Fig 1).

**Table 2:** Summary statistics for sequence quality and alignment information of breast muscle samples in two groups.

	Ammonia3_1	Ammonia3_2	Ammonia25_1	Ammonia25_2
**Group**	Ammonia3	Ammonia3	Ammonia25	Ammonia25
**Raw reads**	73,988,890	70,579,138	87,417,108	87,567,900
**Raw bases**	9,248,611,250	8,822,392,250	10,927,138,500	10,945,987,500
**Clean reads**	70,089,536	66,879,214	82,649,276	82,713,992
**Clean bases**	8,757,428,504	8,356,297,248	10,326,600,930	10,334,744,020
**Valid ratio (base)**	94.68%	94.71%	94.50%	94.41%
**Q30 (%)**	94.75%	94.79%	94.64%	94.58%
**GC content (%)**	51.50%	51.50%	51.50%	51.50%
**Total mapped reads**	49,313,882	47,427,718	59,205,224	58,723,920
**Uniquely mapped reads**	45,818,693	44,268,019	54,687,437	54,278,215
**Multiple mapped reads**	3,495,189	3,159,699	4,517,787	4,445,705
**Mapping rate (%)**	70.36%	70.92%	71.63%	71.00%

**Fig 2:**
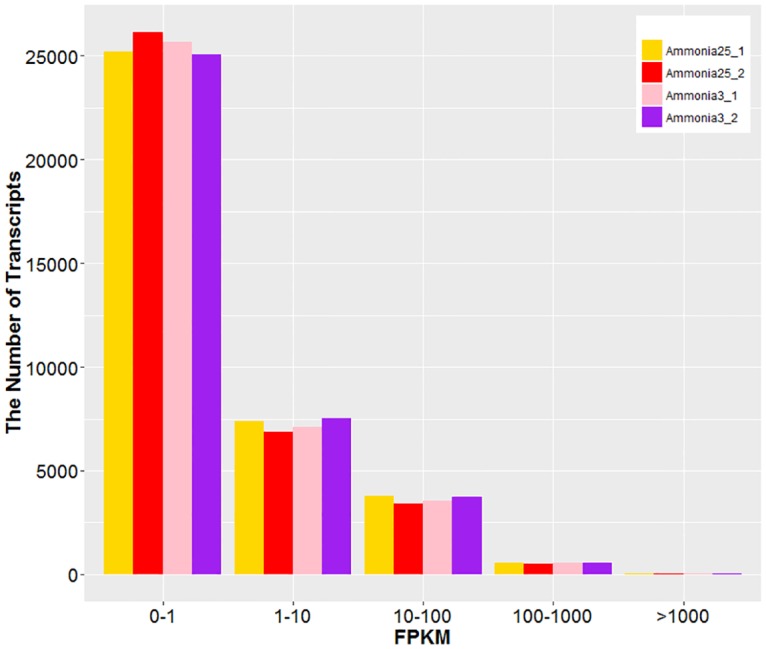
Distribution of expression level (in FPKM) of transcripts.

The relative expression of genes is normalized as fragments per kilobase of exon model per million mapped reads (FPKM), which is proportional to the number of cDNA fragments originated from the gene transcript. In total, 36977 transcripts of breast muscle were identified, of these transcripts, approximate 70% were low expression ones (Fig 2). The lowest limit of gene expression value is set to be 0.6 FPKM in at least one of the 4 samples. According to this limit, 11,095 genes are identified as being expressed in the breast muscle tissues (S3 Table). The correlation analysis based on the gene expression profiles revealed that the correlations among two samples per group both were greater than 0.92 (S1 Fig).

### Differentially Expressed Genes

**Fig 3:**
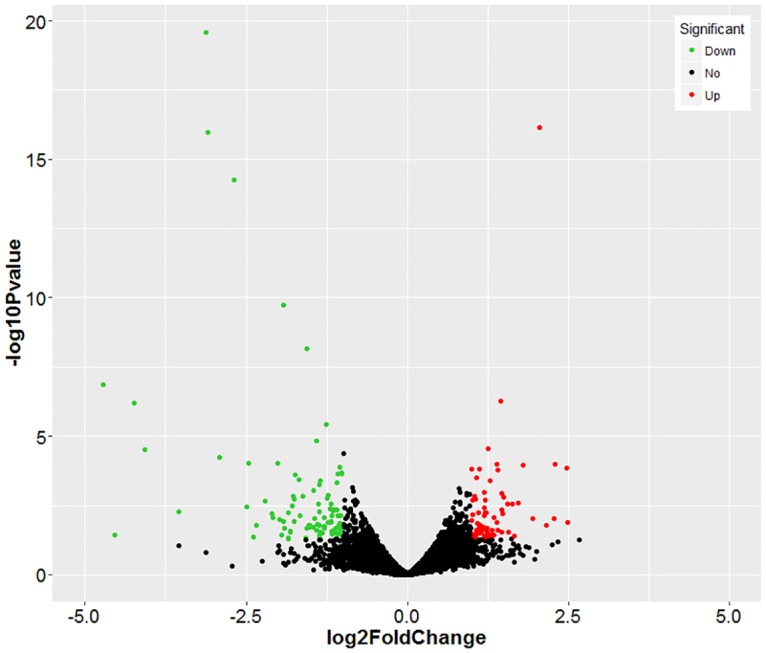
Volcano plot displaying differentially expressed genes between two groups. Red dots (Up) represent significantly up-regulated genes (*P* < 0.05, fold change ≥ 2); green dots (Down) represent significantly down-regulated genes (*P* < 0.05, fold change ≤ 0.5); black dots (No) represent insignificantly differentially expressed genes.

**Fig 4:**
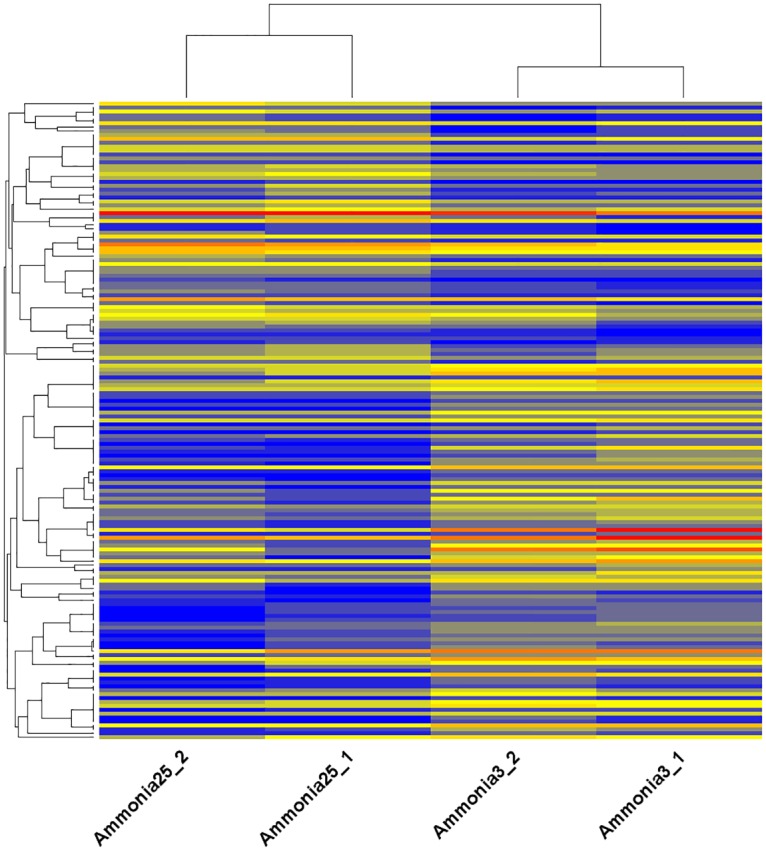
Heat map of differentially expressed genes between two groups.

Differential gene expression analysis between two groups showed that 163 genes were significantly differentially expressed (fold-change ≥2 or ≤ 0.5 at *P* < 0.05), including 67 up-regulated and 96 down-regulated genes (Fig 3, S2 Fig, S3 Table). The top ten up- and down-regulated genes are listed in Table 3. The fold changes induced by high concentrations of ammonia ranged from -26.3 to 5.6 (Fig 4 and Table 3).

**Table 3:** Top ten up- and down-regulated genes in treatment group compared to control group.

Gene ID	Gene symbol	Fold change	*P*-value
NM_001277411.1	*CA3*	-26.29	1.32E-07
NM_001167752.2	*MB*	-23.34	3.82E-02
XM_416332.4	*MYBPC1*^*1*^	-18.90	6.41E-07
XM_004937841.1	*MYBPC1*^*2*^	-16.88	3.11E-05
NM_001113709.1	*MYH1C*	-11.67	5.48E-03
XM_416965.4	*METTL21C*	-8.76	2.58E-20
XM_004940680.1	*FHL1*^*1*^	-8.52	1.06E-16
XM_004946064.1	*MYH1G*	-7.56	5.92E-05
XM_001234113.3	*FHL1*^*2*^	-6.45	5.52E-15
NM_205446.1	*TPM2*	-5.62	3.49E-03
XM_418427.4	*KHDRBS3*	3.16	4.04E-02
XM_004934600.1	*AGPAT3*	3.30	2.50E-03
NM_205028.1	*CNTFR*	3.47	1.08E-04
NM_001030966.1	*INSIG1*	3.86	9.10E-03
XM_417174.4	*DCUN1D5*	4.13	6.98E-17
XR_211672.1	*LOC101748992*	4.46	1.59E-02
NM_001031288.1	*DHCR24*	4.82	9.32E-03
NM_001007477.3	*LOC396531*	4.88	9.83E-05
XM_004943064.1	*42436*	5.52	1.47E-04
NM_001012846.1	*ACSBG2*	5.61	1.25E-02

*CA3*, carbonic anhydrase III, muscle specific; *MB*, myoglobin; *MYBPC1*^*1*^, myosin binding protein C, slow type, transcript variant X2; *MYBPC1*^*2*^, myosin binding protein C, slow type, transcript variant X1; *MYH1C*, myosin, heavy chain 1C, skeletal muscle; *METTL21C*, methyltransferase like 21C; *FHL1*^*1*^, four and a half LIM domains 1, transcript variant X4; *MYH1G*, myosin, heavy chain 1G, skeletal muscle (similar to human myosin, heavy chain 1, skeletal muscle, adult); *FHL1*^*2*^, four and a half LIM domains 1, transcript variant X1; *TPM2*, tropomyosin 2 (beta); *KHDRBS3*, KH domain containing, RNA binding, signal transduction associated 3; *AGPAT3*, 1-acylglycerol-3-phosphate O-acyltransferase 3; *CNTFR*, ciliary neurotrophic factor receptor; *INSIG1*; insulin induced gene 1; *DCUN1D5*, DCN1, defective in cullin neddylation 1, domain containing 5 (S. cerevisiae), transcript variant X2; *LOC101748992*, uncharacterized LOC101748992; *DHCR24*, 24-dehydrocholesterol reductase; *LOC396531*, parvalbumin; *42436*, membrane-associated ring finger (C3HC4) 7, E3 ubiquitin protein ligase; *ACSBG2*, acyl-CoA synthetase bubblegum family member 2.

### GO Annotations and Pathway Analysis

**Fig 5:**
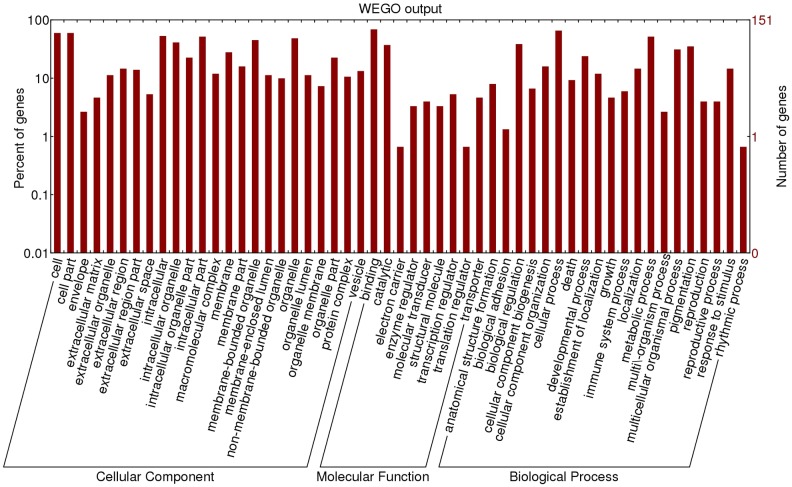
GO analysis of differentially expressed genes in breast muscle from two groups. The number of genes for each GO annotation is shown in right axis, and the proportion of genes for each GO annotation is exhibited in left axis.

**Fig 6:**
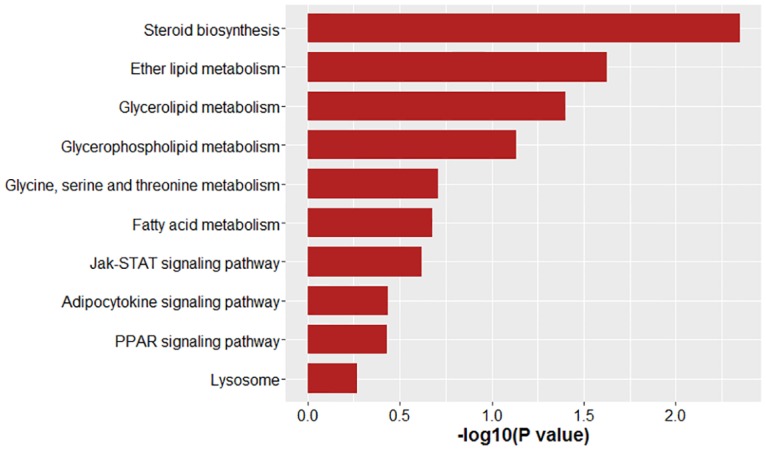
KEGG analysis of enriched pathways of differentially expressed genes between two groups.

In total, 151 differentially expressed genes (DEGs) were assigned to Gene Ontology (GO) terms on the basis of GO annotation. GO enrichment analysis was performed from three aspects including cellular component, molecular function and biological process. Among these categories, most DEGs were enriched in the "cellular component" category (Fig 5). Within the "cellular component" category, "cell" and "cell part" were the most dominant subcategories. Regarding the "molecular function" category, the four most abundant subcategories were "binding", "catalytic", "transcription regulator" and "transporter". As for the "biological process" category, most DEGs were assigned to "cellular process", "metabolic process" and "biological regulation". The following genes involved in these GO term were found to be enriched: *MSTN*, *COL15A1*, *MFAP5*, *HTRA3*, *FBN1*, *TNXB*, *LSS*, *IL15*, *ELN*, *HSD17B7*, *DHCR24*, *ISPD*, *MVD* (Table 4). Then, the DEGs were annotated using KEGG to identify enriched pathways (Fig 6). Pathways that were significantly enriched included steroid biosynthesis, ether lipid metabolism and glycerolipid metabolism (*P* < 0.05).

**Table 4:** Enriched KEGG pathways and GO terms of DEGs in the breast muscle of broilers.

Category	Term	Count	Enriched genes	*P*-value
GO term	cholesterol metabolic process	3	*LSS*, *HSD17B7*, *DHCR24*	0.008
GO term	sterol metabolic process	3	*LSS*, *HSD17B7*, *DHCR24*	0.010
GO term	lipid biosynthetic process	4	*LSS*, *DHCR24*, *ISPD*, *MVD*	0.025
GO term	extracellular region part	7	*TNXB*, *MFAP5*, *COL15A1*, *IL15*, *ELN*, *MSTN*, *FBN1*	0.007
GO term	extracellular region	8	*HTRA3*, *TNXB*, *MFAP5*, *COL15A1*, *IL15*, *ELN*, *MSTN*, *FBN1*	0.036
KEGG pathway	Steroid biosynthesis	3	*LSS*, *HSD17B7*, *DHCR24*	0.004
KEGG pathway	Ether lipid metabolism	3	*AGPAT2*, *AGPAT6*, *AGPAT3*	0.024
KEGG pathway	Glycerolipid metabolism	3	*AGPAT2*, *AGPAT6*, *AGPAT3*	0.040

*LSS*, lanosterol synthase (2,3-oxidosqualene-lanosterol cyclase); *HSD17B7*, hydroxysteroid (17-beta) dehydrogenase 7; *DHCR24*, 24-dehydrocholesterol reductase; *ISPD*, isoprenoid synthase domain containing; *MVD*, mevalonate (diphospho) decarboxylase; *HTRA3*, HtrA serine peptidase 3; *TNXB*, tenascin XB; *MFAP5*, microfibrillar associated protein 5; *COL15A1*, collagen, type XV, alpha 1; *IL15*, interleukin 15; *ELN*, elastin; *MSTN*, myostatin; *FBN1*, fibrillin 1; *AGPAT2*, 1-acylglycerol-3-phosphate O-acyltransferase 2; *AGPAT6*, 1-acylglycerol-3-phosphate O-acyltransferase 6; *AGPAT3*, 1-acylglycerol-3-phosphate O-acyltransferase 3.

### Validation of Genes of Differential Abundance

**Fig 7:**
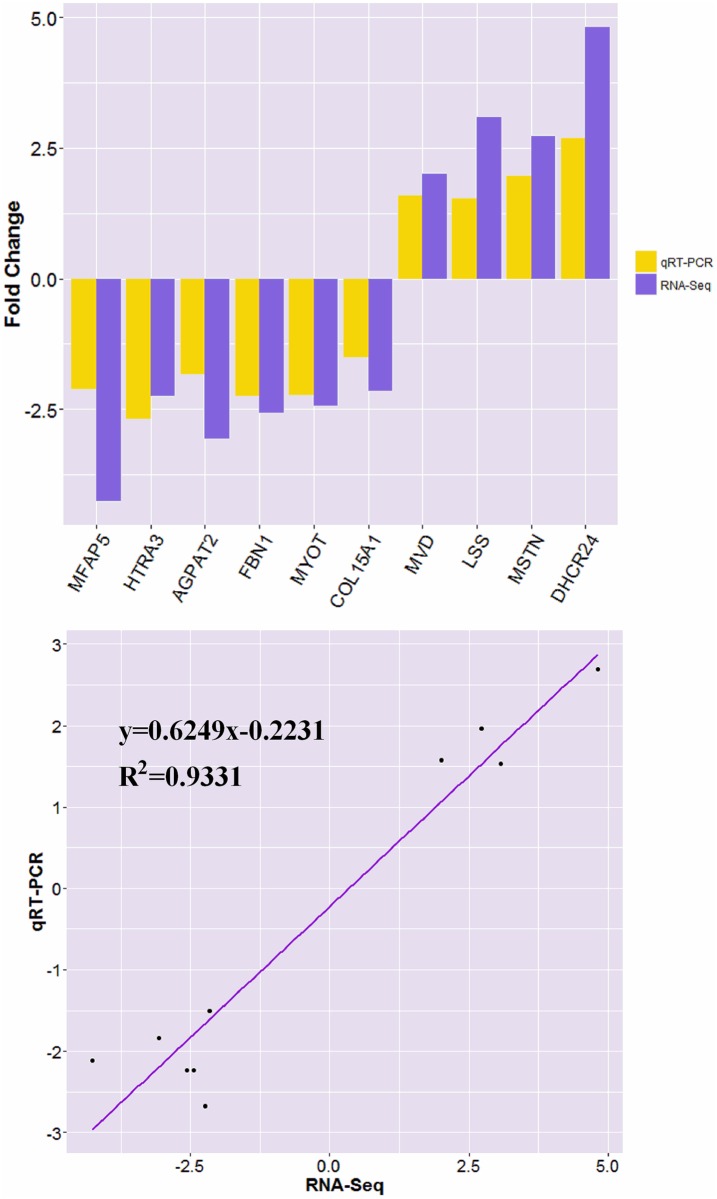
qRT-PCR analysis of differentially expressed genes from breast muscle of AA broilers. *MSTN*, myostatin; *MFAP5*, microfibrillar associated protein 5; *COL15A1*, collagen, type XV, alpha 1; *FBN1*, fibrillin 1; *DHCR24*, 24-dehydrocholesterol reductase; *HTRA3*, HtrA serine peptidase 3; *MVD*, mevalonate (diphospho) decarboxylase; *LSS*, lanosterol synthase (2,3-oxidosqualene-lanosterol cyclase); *AGPAT2*, 1-acylglycerol-3-phosphate O-acyltransferase 2; *MYOT*, myotilin. *GAPDH* was used as an internal control, data are presented as fold change (n = 3 per group).

To validate the differentially expressed genes of breast muscle between control and treatment groups obtained by transcriptome, ten genes were randomly selected and their expression levels were quantified using qRT-PCR (Fig 7). The RNA-Seq results indicated that the expression of *MFAP5*, *AGPAT2*, *FBN1*, *MYOT*, *HTRA3* and *COL15A1* were down-regulated in treatment group by two to four-fold, the expression of *MVD*, *LSS*, *DHCR24* and *MSTN* were up-regulated in treatment group by two to five-fold. qPCR results also found the similar down- or up-regulated trend in the expression of these genes. Therefore, the qRT-PCR results validate the findings by high throughput RNA sequencing.

## Discussion

High concentrations of atmospheric ammonia are a serious threat to the health and production in broiler industry. Many past studies have exposed animals to extremely high concentrations of ammonia, far higher than are present in commercial poultry buildings [10,14]. This work was conducted to determine the effect on performance of broiler chickens exposed to more realistic concentrations of aerial ammonia. In this report, the results revealed an insignificant adverse effect of ammonia on ADG, ADFI and FCR of broiler chickens, despite 21 days of chronic exposure to 25 ppm levels of ammonia, which was consistent with the research of Yahav [31], and inconsistent with Caveny, Johnson, Charles and Payne [17,25,32] about the effect of ammonia on growth performance in broilers. The deleterious effects of aerial ammonia on weight gain, feed intake and FCR of broilers have been reviewed by many scientific researchers [4,33,34]. To the truth, the effect of ammonia on broilers performance has not been well established until now. These different results may be due to the different breeds and developmental stages of broiler chickens, difference in exposure time and levels of ammonia, and even different ways of management and climatic conditions, and so on [8].

The adverse effect of atmospheric ammonia on broiler chickens was mainly concentrated on respiratory system. In the current study, we focus on the breast muscle tissue, because breast meat is the prime part of carcass and breast meat yield is of practical importance to the profitability of broiler production [23,35,36]. In agreement with the results reported by Mile et al. and Reece et al. [10,14], our data showed that broilers in Ammonia25 group had lower beast muscle percentage than those in Ammonia3 group. In addition, the slaughter rate of broilers also declined which is an important parameter in the production of broiler meat [37]. In poultry industry, broilers' production and profitability were affected not only when exposed to moderate to heavy concentrations of atmospheric ammonia but also relatively low concentrations of ammonia.

Previous reports demonstrated that ammonia exposure decreased the body weight and breast meat yield, but the mechanism was not included [10]. Illumine transcriptome profiling as an efficient, fast tool is now widely used in stock raising [38]. In the present study, high throughput RNA sequencing technology was used to dig the probable candidate genes and processes in breast muscles that result from aerial ammonia exposure. Our results revealed that several key DEGs participating muscle development were identified in enriched GO terms. This may provide an explanation for lower breast muscle ratio in broilers because of ammonia exposure.

Myostatin (*MSTN*) also known as growth and differentiation factor-8 (*GDF-8*), mainly expressed in skeletal muscle, is a dominant inhibitor of skeletal muscle development, differentiation and growth [39]. Mutations in the coding region of *MSTN* are known to cause an increased muscle mass ("double muscling") phenotype in several mammals, including mice, dogs, cattle, sheep and humans [40,41]. On the contrary, transgenic mice overexpressing *MSTN* had decreased muscle mass [42]. In poultry, Kim et al. reported that broilers from eggs that had the *MSTN* antibody injected into the yolk had significantly heavier body and muscle mass than the controls, no matter in male or female birds [43,44]. Consistent with previous research, our data indicated that upregulated expression of *MSTN* negatively affected meat yield of broilers in Ammonia25 group than those in Ammonia3 group.

As for the mechanism of high concentrations of ammonia would upregulate the expression of *MSTN*, the work of Qiu et al. may provide a confirm evidence. Their research revealed that hyperammonemia stimulated *MSTN* expression in murine models, and maybe in a NF-κB-dependent manner [45]. Surprisingly, in broilers embryos, the induced hyperammonemia model showed lower myostatin expression [46]. This result may be due to the relatively short-term exposure to hyperammonemia compared with the previously reported rat model with a long-term hyperammonemic condition or the murine model with induced hyperammonemia [45–47]. The experimental condition and design in our trial was more similar to the report of Qiu et al. Therefore, according to these previous research, we can deduce that broilers exposed to high levels of ammonia would and have a negative effect on breast muscle growth and development via upregulating the expression of *MSTN*. Whether ammonia stimulated *MSTN* expression through a NF-κB-dependent manner in broilers is still need further research.

## Conclusions

In summary, this study has analyzed the transcriptome of breast muscle tissue in broiler chickens exposed to high (25 ppm) or low (3 ppm) atmospheric ammonia by RNA sequencing. The RNA sequencing identified a series of differentially expressed genes in muscles, including candidate myostatin gene, with respect to broilers exposed to two different levels of ammonia with significantly different amounts of breast meat percentage. These genes could be of great importance for understanding the molecular basis of skeletal muscle growth stimulated by aerial ammonia in broiler chickens. This study also provides a theoretical foundation for reducing ammonia concentrations, improving air quality and animal welfare.

## Supporting Information

S1 Fig Correlation plots of the reads (in FPKM) for two groups. (TIF)

S2 Fig MA plots displaying differentially expressed genes between two groups. (TIF)

S1 Table Composition of the experimental diet and calculated proximate composition of the diet. (DOCX)

S2 Table Primer sequences and product sizes. (DOCX)

S3 Table The relative expression of transcripts (in FPKM) of breast muscle from broilers in two groups. (XLSX)

S4 Table The differentially expressed genes between two groups. (XLSX)
