# Supplementary figures and images for: Transcriptome Profile Analysis of Breast Muscle Tissues from High or Low Levels of Atmospheric Ammonia Exposed Broilers (Gallus gallus)
Source: PLoS One. 2016 Sep 9;11(9):e0162631. doi: 10.1371/journal.pone.0162631 (PMC5017607; doi:10.1371/journal.pone.0162631)

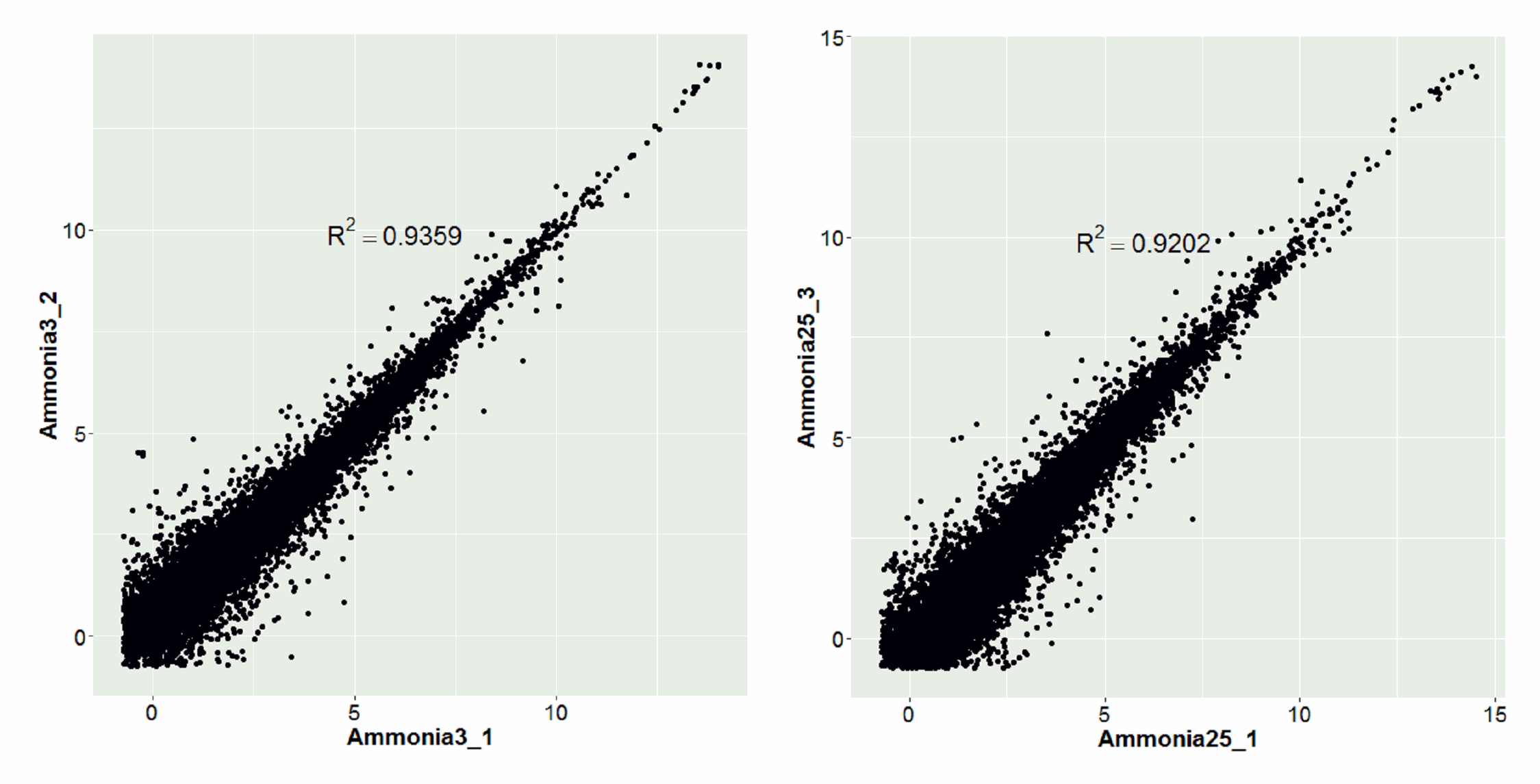

Supplement: S1 Fig — (TIF) [file pone.0162631.s001.tif]

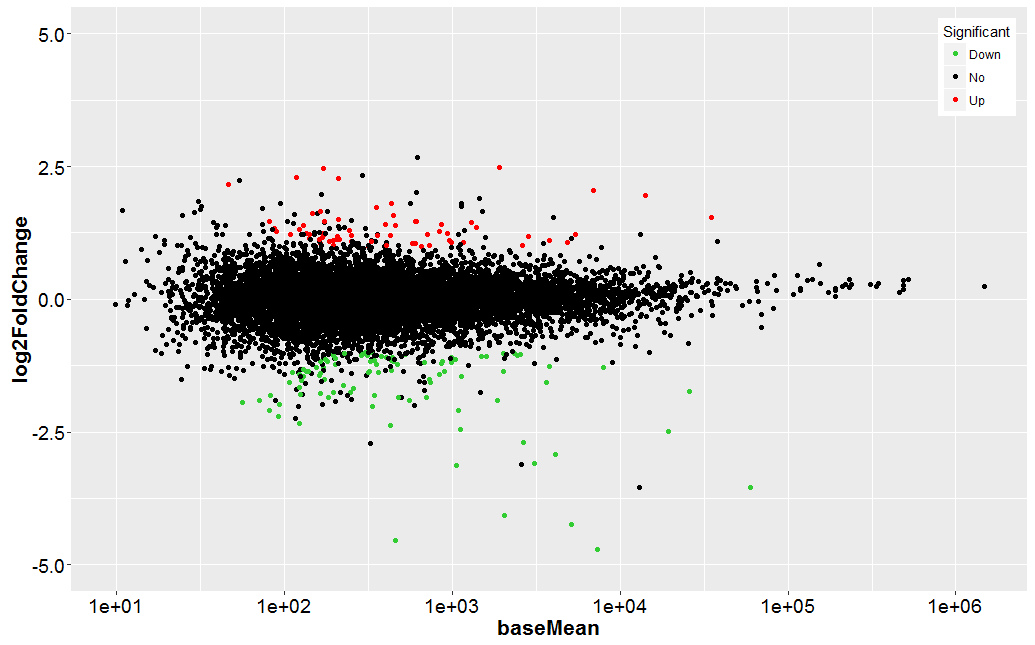

Supplement: S2 Fig — (TIF) [file pone.0162631.s002.tif]
