# Supplementary material for: Transcriptome Profile Analysis of Breast Muscle Tissues from High or Low Levels of Atmospheric Ammonia Exposed Broilers (Gallus gallus)
Source: PLoS One. 2016 Sep 9;11(9):e0162631. doi: 10.1371/journal.pone.0162631 (PMC5017607; doi:10.1371/journal.pone.0162631)
Supplement: S2 Table — (DOCX) [file pone.0162631.s004.docx]

**Table S2 Primer sequences and product sizes**

| **Gene symbol** | **Accession no.** | **Primer sequence** | **Product size (bp)** |
| --- | --- | --- | --- |
| *GAPDH* | NM_204305 | 5'-CACTGTCAAGGCTGAGAACGG-3'  5'-GAGATGATAACACGCTTAGCACCA-3' | 192 |
| *MSTN* | NM_001001461 | 5'-AACGGAATCCCGATGTTGTC-3'  5'-GCTTGGTGTACCAGGTGAGTGT-3' | 162 |
| *AGPAT2* | XM_015279793 | 5'-CCACCAGAGCATCCTCGACAT-3'  5'-CCACCTCCGCCATCACCATC-3' | 179 |
| *COL15A1* | XM_004935077 | 5'-CTCTAGTATCTCCCGAGAAGCG-3'  5'-CCTGCATTACCAACAAATATCCC-3' | 198 |
| *DHCR24* | NM_001031288 | 5'-TTCCTGGTTGCAGCAGAAATAA-3'  5'-TCATCAGTCAAGACTCCCGTCA-3' | 194 |
| *FBN1* | XM_015291935 | 5'-CTGCCAGCAAGACCTGACCT-3'  5'-TGGAGCCCGAGATGGATAGA-3' | 123 |
| *HTRA3* | XM_015285845 | 5'-GGACCTCTAGTTAATCTGGATGGTG-3'  5'-TTTGTTTGTCAAGCGACTCTGTG-3' | 127 |
| *LSS* | NM_001006514 | 5'-CACCTACCTACGTCAGCGAGCCA-3'  5'-CATCCACAAACCAGCGAACCAAC-3' | 131 |
| *MFAP5* | XM_015292825 | 5'-GCAACCTTTATGGCATTGTATCTC-3'  5'-AGGGAACTGCTCCTCACGAC-3' | 192 |
| *MVD* | XM_423130 | 5'-ACGCCACCTGCCTTGACACCTTCC-3'  5'-CCTCACCACCTCCACGAACTCATCCA-3' | 191 |
| *MYOT* | XM_015293961 | 5'-TCTCCCATCCGTTTCCTCTAT-3'  5'-TGATTTGCCTGCTTGACTCTG-3' | 143 |

*MSTN,* myostatin; *MFAP5,* microfibrillar associated protein 5; *COL15A1,* collagen, type XV, alpha 1; *FBN1,* fibrillin 1; *DHCR24,* 24-dehydrocholesterol reductase; *HTRA3,* HtrA serine peptidase 3; *MVD,* mevalonate (diphospho) decarboxylase; *LSS,* lanosterol synthase (2,3-oxidosqualene-lanosterol cyclase); *AGPAT2,* 1-acylglycerol-3-phosphate O-acyltransferase 2; *MYOT,* myotilin. *GAPDH,* glyceraldehyde-3-phosphate dehydrogenase.
